# Review of Synthetic and Hybrid Scaffolds in Cartilage Tissue Engineering

**DOI:** 10.3390/membranes10110348

**Published:** 2020-11-17

**Authors:** Monika Wasyłeczko, Wioleta Sikorska, Andrzej Chwojnowski

**Affiliations:** Nałęcz Institute of Biocybernetics and Biomedical Engineering, Polish Academy of Sciences, Trojdena 4 str., 02-109 Warsaw, Poland; wsikorska@ibib.waw.pl (W.S.); achwoj@ibib.waw.pl (A.C.)

**Keywords:** cartilage tissue engineering, articular cartilage, scaffolds, scaffold obtaining methods, materials for scaffolds, scaffold requirements, synthetic and hybrid scaffolds, chondrocytes, mesenchymal stem cells, tissue engineering, regenerative medicine

## Abstract

Cartilage tissue is under extensive investigation in tissue engineering and regenerative medicine studies because of its limited regenerative potential. Currently, many scaffolds are undergoing scientific and clinical research. A key for appropriate scaffolding is the assurance of a temporary cellular environment that allows the cells to function as in native tissue. These scaffolds should meet the relevant requirements, including appropriate architecture and physicochemical and biological properties. This is necessary for proper cell growth, which is associated with the adequate regeneration of cartilage. This paper presents a review of the development of scaffolds from synthetic polymers and hybrid materials employed for the engineering of cartilage tissue and regenerative medicine. Initially, general information on articular cartilage and an overview of the clinical strategies for the treatment of cartilage defects are presented. Then, the requirements for scaffolds in regenerative medicine, materials intended for membranes, and methods for obtaining them are briefly described. We also describe the hybrid materials that combine the advantages of both synthetic and natural polymers, which provide better properties for the scaffold. The last part of the article is focused on scaffolds in cartilage tissue engineering that have been confirmed by undergoing preclinical and clinical tests.

## 1. Introduction

Most human tissues and organs have a limited capacity to properly self-regenerate. Moreover, they are often exposed to damage as a result of injuries, accidents, and various diseases involving tissue dysfunction or devastating deficits [1,2]. Many surgical strategies have been developed to ameliorate these problems, including the transplantation of artificial substitutes, such as joint prostheses, heart valves, kidneys, or even tissues and organs [3,4,5]. Unfortunately, the main obstacles for organ transplantation are the deficit of donor organs and the necessity of lifelong immunosuppression. Nonbiological components can cause particular problems, such as a lack of biocompatibility, the development of serious infections, and limited durability [6,7]. Therefore, regenerative medicine including tissue engineering (TE) is a promising domain of research that can offer not only tissues and organs for transplantation but can also provide new perspectives for the treatment of many diseases [8]. This is due to the combination of biological sciences and material engineering methods enabling the development and acquisition of biological substitutes [2,9]. At present, regenerative medicine offers methods for treating various tissues, including the skin, musculoskeletal tissue, the liver, gastrointestinal tissue, nervous system tissue, and cardiovascular tissue [9,10,11,12] and can even treat diseases such as diabetes [13,14]. Currently, scaffolds are increasingly popular substitutes in TE. Scaffolds can be used for in vitro cultures of appropriate cells, which can be implanted into the body as a bio-implant after a suitable amount of time and can also be transplanted directly into the organism as a medium for the colonization of host stem cells [15,16,17,18]. Scaffolds should be three-dimensional with a network of an interconnected pore structure and tunable sizes that depend on the kinds of cells. A scaffold needs to be biocompatible and provide appropriate mechanical stability and shape properties to resist stresses during cultivation and after being implanted into the body [19,20,21].

An example of a tissue with limited regenerative capacity is cartilage, due to its lack of vascularization and innervations [22]. Scientists and doctors are still looking for an effective method to regenerate cartilage, and the most promising method is to use scaffolds [22,23,24,25].

This review article presents a general description of articular cartilage, the problems faced by this organ, and the current methods for its treatment. Next, the requirements and materials for scaffolds in cartilage engineering are presented, along with general methods for their preparation. In this section, the requirements for scaffolds using chondrocytes and stem cells are also highlighted. The last section of the article presents scaffolds made of synthetic polymers and their combinations with natural materials (hybrid scaffolds). This work focuses on research fromthe last decade, taking into consideration scaffolds that are currently under development as well as those that have undergone or are undergoing clinical research.

## 2. Articular Cartilage and Clinical Strategies for Treatment

Cartilage is a skeletal connective tissue classified into three types, for which hyaline is necessary to enable proper movement [23,26]. Cartilage is still a still problem for regenerative medicine because there is no effective treatment for its reconstruction. Currently, supportive treatment methods are used. Articular cartilage and its general treatment methods are briefly discussed in this section.

### 2.1. Articular Cartilage: Characteristics, Roles, Joint Diseases, and Traumatic Lesions

Articular cartilage (AC) is a type of hyaline cartilage. It is a hard and elastic tissue located between the bones. AC is composed of spheroid cells called chondrocytes and is 10 to 13 μm in diameter [23]. These cells constitute about 2% of the total volume of AC and produce an extracellular matrix (ECM) that is rich, among others, in collagen type II and proteoglycans. As a solid phase, AC is porous and permeable. The main component of the fluid phase of AC is water with inorganic ions such as sodium, chloride, and potassium. Cells are protected by the surrounding ECM from damaging forces. Cartilage is an avascular and aneural tissue, so it has no ability to transfer nutrients to cells (ECM helps transfer nutrients to chondrocytes via diffusion from the synovial fluid). This means that cartilage does not have a self-repair ability, which is why the role of doctors and scientists in cartilage tissue engineering/regenerative medicine is important. The principle functions of AC are, among others, to protect the ends of the bones from damage caused by movement (acting as a shock absorber). AC provides the mechanical ability to withstand loads and impacts and also provides a low-friction gliding surface. Trauma, an unhealthy lifestyle, traffic accidents, or various diseases (e.g., gene mutations and autoimmune disorders) can damage the cartilage, causing pain, movement limitations, stiffness, swelling, and even disability [23,26,27,28,29]. Examples of joint-damaging diseases include obesity and osteoporosis. This leads to an abrasion of the cartilage, which loses its elasticity and resistance to friction. Initially, this process is painless due to the lack of innervation and blood supply in the cartilage (it cannot be regenerated). Cartilage wear involves slow joint death, which is a consequence of aging and the accumulation of injuries from youth. Many people with knee ligament or meniscus damage have damaged cartilage after a few years [28,30]. Another type of cartilage damage occurs due to the disease osteochondritis dissecans, in which the bone dies and is secreted into the joint with the cartilage covering it. This bone and cartilage can then fracture and become loose [31]. Next, juvenile idiopathic arthritis (JIA) is the most common type of childhood arthritis. This autoimmune disease is a chronic inflammatory process that damages the articular cartilage, induces bone epiphysis, and is responsible for extra-articular symptoms and systemic complications. This disease can occur at any stage of developmental age and its very wide symptomatology creates diagnostic problems, especially in the initial stages of the disease development [29,32]. The most common disease connected with articular cartilage defects is osteoarthritis (OA). This is the most common musculoskeletal disorder resulting from the degradation of cartilage and leads to a poor quality of life and disability. It can affect any joints in the body, including those in the knees, hips, spine, or fingers. Factors that can affect the development of this disease include genetic factors, obesity, inflammation, trauma, occupational factors, or metabolic syndrome. Moreover, OA progresses with age and mainly affects women. Without treatment, no recovery can be achieved [30,33,34,35,36].

### 2.2. Treatment Methods for Cartilage Regeneration

Despite much research on the matter, there are no effective treatments for OA. Current clinical methods focus mostly on pain treatment and are not satisfactory [37]. Many clinical techniques to repair/regenerate cartilage are known. Which method will be used depends on factors such as the area of damage, the depth, location, associated damage, chronicity, and age, as well as the physical activity of the patient. The depth or degree of cartilage damage is a key factor that determines the choice of treatment method. In classifying the degree of damage, many divisions are used to describe both the depth and the area of damage. The most widely used system is the Outerbridge classification, which takes into account size and depth (Table 1) [38,39].

In grades I and II, conservative treatments, such as patient education, reduction of BMI, rehabilitation, or the application of pharmacological treatment and dieting, usually give good results. Surgical interventions are recommended for grades III or IV. The most commonly used surgical methods are the microfracture (MF) method, chondroplasty surgery, osteochondral transplantation, and mosaicplasty, as well as cell-based approaches, such as autologous chondrocyte implantation (ACI). The microfracture technique is used much more frequently than other techniques but is not satisfactory. In recent years, the progress of cartilage tissue engineering has provided great hope for the regeneration of damaged cartilage. In every case, the doctor must decide which method is appropriate to choose. Each method has its own indicators and limitations, as well as advantages and disadvantages. The most appropriate management should be implemented at every stage of cartilage damage. The more extensive and serious the damage is, the more difficult and complicated the therapy will be, and the lower the chance of a full recovery [22,24,25,40].

MF is a safe, minimally invasive, and cheap method for cartilage repair. MF is a subchondral bone marrow stimulation method where a blood clot fills the defect. This provides a suitable environment for tissue regeneration. Unfortunately, the MF technique promotes regeneration to fibrocartilage tissue with inferior biomechanical properties compared to hyaline cartilage [37,41].

**Figure 1:**
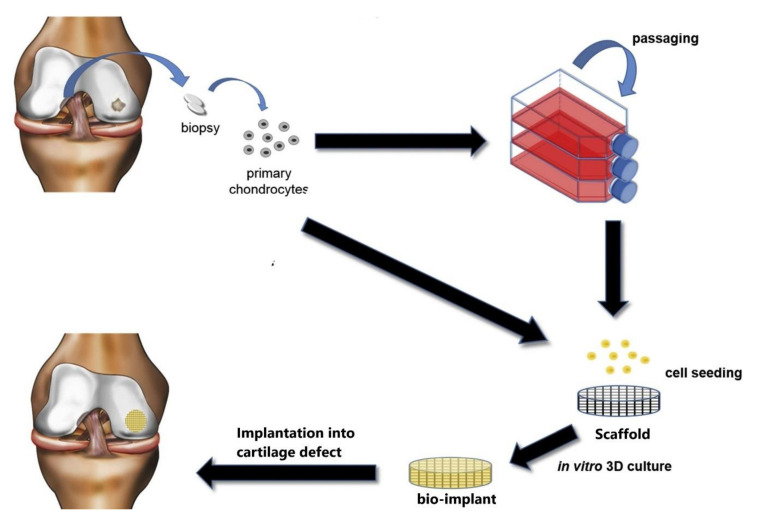
General schema of the autologous chondrocyte implantation (ACI) method with a 3D scaffold. This schema was modified according to a previous article [44].

**Figure 2:**
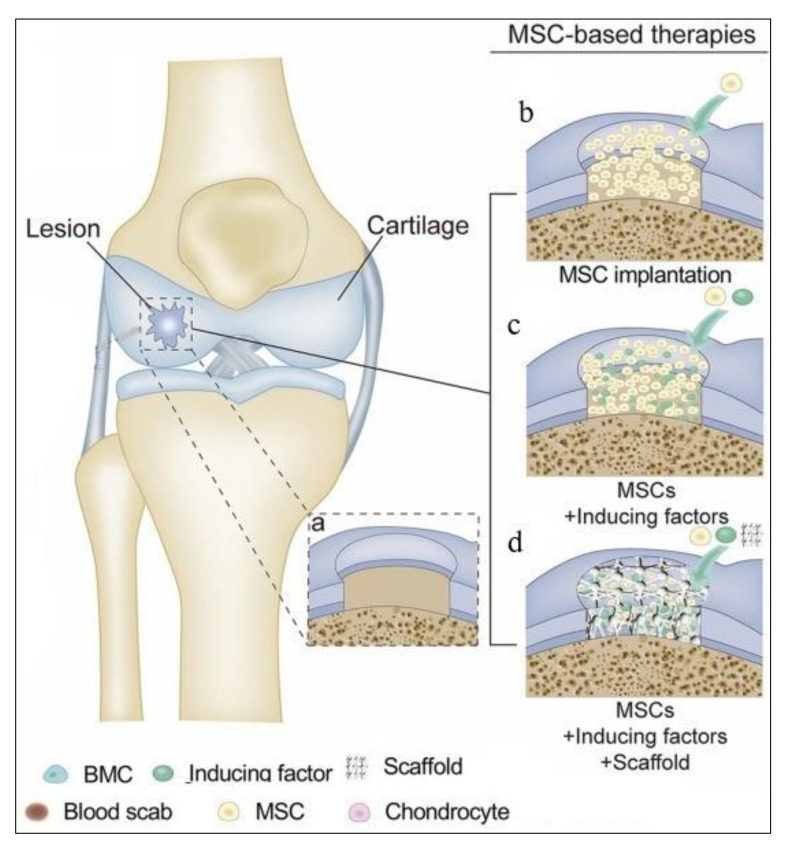
Cartilage repair methods via mesenchymal stem cell (MSC)-based therapies: (**a**) full-thickness cartilage injury; (**b**–**d**) therapies using MSCs and appropriate additives. The schema was modified from a previous article [53].

More promising methods for the treatment of chondral lesion are cell-based approaches. These techniques enable the implantation of articular chondrocytes (ACs) from the patient in place of the defect. ACI with or without a scaffold is used in routine clinical practice. Three-dimensionalscaffolds serve as a temporary matrix for chondrocytes isolated from a healthy non-load-bearing area of the patient’s cartilage. Generally, the therapeutic cells are cultured in vitro on scaffolds. Then, a bio-implant is transplanted into the tissue defect. The scaffold is gradually degraded along with cartilage formation (Figure 1) [22,25,40,42,43]. The schema in Figure 2 presents the ACI method with a scaffold, showing a version where ACs can be passaged to multiply them or placed directly in the scaffold for an in vitro culture.

Compared to MF, ACI allows the repair of larger cartilage defects. Moreover, studies indicate better results for ACI compared to MF [40,45]. Unfortunately, no current treatment for articular cartilage repair has recreated native hyaline cartilage. Current approaches reconstruct fibrocartilage, which is susceptible to further damage. However, combining different approaches, including advanced scaffolds, growth factors, or alternative cell types, such as mesenchymal stem cells (MSCs), provides an alternative for obtaining an effective cartilage treatment method. MSCs can be obtained from different sources, such as adipose tissue and bone marrow with the potential to differentiate into ACs. In addition, this approach can avoid the invasion of the joint for the initial harvesting of ACs [22,25,40,43,46,47,48,49,50,51].

Scaffolds can also serve as carriers of chondrogenic cells, MSCs, and the bioactive factors influencing chondrocyte growth and differentiation (growth factors) or their combinations. Recent treatments have indicated possibilities to regenerate articular cartilage using the surgical implantation of MSCs into articular cartilage lesions (Figure 2). Scaffolds for MSCs should meet the appropriate parameters described in Section 3.1.

Thanks to advances in medicine, it is now possible to collect stem cells not only from umbilical cord blood, endometrium, or bone marrow [46,47,49] but also from the adult tissues of each organism, especially from adipose tissue (AT) [47,52,53]. The main advantage of AT is its availability and abundance. Studies show that there are many times more stem cells in AT than in bone marrow. MSCs obtained from their own AT have a significant advantage over bone marrow cells due to their availability and large number. The collection procedure itself is also less painful and invasive for the patient [47,52,54,55].

In clinical methods, growth factors for ACs and MSCs are helpful. Studies have shown the effects of growth factors on chondrogenesis and the maintenance of the correct phenotypes of cells. These growth factors can be added to the medium or scaffold during cultivation. Polypeptide mediators, such as transforming growth factor β (TGF-β), insulin-like growth factor (IGF), and fibroblast growth factor (FGF), stimulate the proliferation of cartilage cells and stabilize their phenotypic expression and chondrogenesis. It has been shown that the therapeutic potential of growth factors in the process of cartilage regeneration is significant. Under the influence of these factors, tissue is formed, the histological structure and biochemical properties of which are similar to hyaline cartilage. Moreover, they hasten the healing of the defect and increase the content of type II collagen compared to I [46,53,56].

## 3. Scaffold for Articular Cartilage Repair: Requirements, Materials, and Method for Obtaining

The role of scaffolds in cartilage tissue engineering is to provide a suitable environment for cells and guarantee success in the tissue regeneration process; this is possible by providing an environment similar to native articular cartilage. Therefore, scaffolds must possess adequate parameters, such as correct architecture, biocompatibility, degradability, or specific chemical and physical properties. This can be achieved through the choice of appropriate materials, additives, such as pore precursors, and manufacturing methods [17,57].

This section presents the requirements for scaffolds in tissue cartilage engineering, the available materials, and techniques for obtaining said scaffolds.

### 3.1. Requirements for Scaffolds

**Figure 3:**
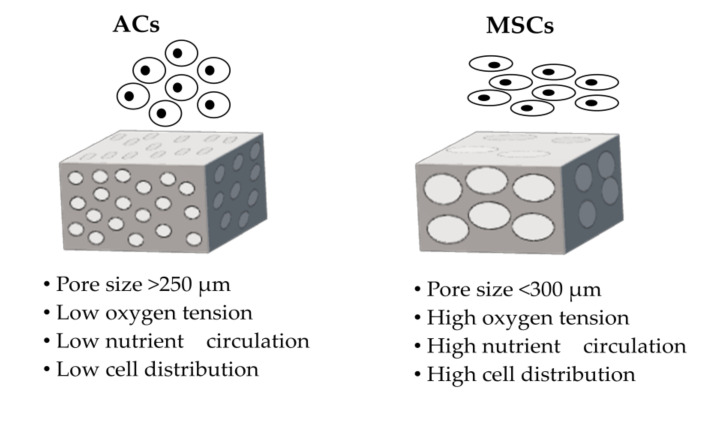
General schematic demonstration of the scaffold properties for the appropriate growth of articular chondrocytes (ACs) and mesenchymal stem cells (MSCs).

Scaffolds for cartilage tissue engineering should provide an appropriate environment and enable cell adhesion, migration, and development by having an appropriate architecture, controlled degradability, adequate mechanical parameters, and good biocompatibility. Many structural features, including porosity, pore size, interconnectivity, and permeability, play a meaningful role in AC development and cartilage regeneration [15,17,51,57]. A three-dimensional design for scaffolds is necessary to prevent the dedifferentiation of chondrocytes into fibroblast-like cells or the chondrogenesis of MSCs [50,58,59,60]. Chondrocytes cultivated on flat surfaces lose their ability to produce particular proteins that are necessary to the formation of hyaline cartilage [61,62]. A highly porous membrane with an interconnected macro-pore network can improve cell seeding, cell migration, cell development, and tissue ingrowth [19,50]. Moreover, the membrane’s structure should be micro-porous to ensure the diffusion of oxygen, nutrients, and metabolism products. Regulated and controlled biodegradation are relevant to the formation of newly regenerating tissue cartilage and mainly depend on the materials used. The products released during degradation should be non-toxic to the body and easily removable. Scaffolds should maintain appropriate parameters in their stiffness, strength, and flexibility, conducive to integration and further tissue development. These parameters are important during cultivation and especially after implantation into the body due to the conditions in the knee [15,19,44,50,51,57,58,59,63,64,65]. Moreover, the parameters of scaffolds should be adapted to the types of cells. The sizes of the macro-pores must also be properly adjusted to the types of cells [15,17,19,64]. Pore sizes of about 150–250 µm are desirable for ACs, whereas large pore sizes of more than 300 µm are adequate for MSCs. Using the right pore sizes will support cell proliferation, the preservation of an appropriate phenotype, and chondrogenic differentiation (Figure 3) [17,20,64,66,67,68,69,70].

To obtain an appropriate scaffold structure depends on the relevant methods, materials, and pore precursors. One way to obtain suitable pores is the use of nonwovens produced by the electrospinning method. Depending on the nonwoven used, pores of 150 µm or greater can be produced [71,72]. To obtain information on the sizes of the pores in scaffolds, specialized programs can be used. One such program is MeMoExplorer™, an advanced membrane morphology software that analyzes SEM images. This software enables the contouring of pores and the measurement of their surfaces. These pores are partitioned into various size-classes, and measurements of the total areas (porosity coefficients) are provided [73,74]. Moreover, using pore precursors such aspoly(vinyl pyrrolidone) or poly(ethylene glycol) can improve the hydrophilicity and mechanical properties of membranes. This is important due to the hydrophobic nature of most synthetic polymers used for scaffold manufacturing [67,75]

Thus, physical parameters such as stiffness, the structures of scaffolds (e.g., pore size, interconnection, and porosity), and culture conditions are important for the fate of the cells. The different conditions for ACs and MSCs are presented above.

### 3.2. Materials Intended for Scaffolds

Materials for scaffolds should be biocompatible, exhibit adequate mechanical parameters, and be biodegradable into non-toxic and non-inflammatory components in the host organism. These materials should also be resistant to the conditions in the body, such as pH and body temperature. Therefore, appropriate materials for the production of scaffolds should be selected. Such materials can be made of synthetic or natural polymers or a combination of both (i.e., hybrid materials (hybrid)) [17,25,51,57,64,76]. Natural materials such as collagen [64,69,77], hyaluronic acid (HA) [78,79], chitosan (CH) [80,81], chondroitin sulfate (CS) [82,83], and fibrin [25,84,85] are widely used in the production of scaffolds for cartilage regeneration. These materials are characterized by their high biocompatibility and bioactivity. Due to their origins, these materials have properties similar to those of native tissues, and most of such materials naturally occur in the human body. These materials support cell attachment and stimulate the production of the ECM. Unfortunately, natural materials have disadvantages. Because of their rapid hydrolysis, natural materials quickly lose their properties suitable for the scaffold structure. Their low mechanical stability is also not adequate to support cells, and their products are thus insufficient for the regeneration of tissue. Moreover, the methods for obtaining such materials are limited due to the low resistance of natural polymers to changes in process parameters, such as high temperatures [25,50,51,57,59,64,76,86].Synthetic polymers, such as poly(ethylene glycol) (PEG) [87,88], polycaprolactone (PCL) [89,90], polylactic acid (PLA) [87,91,92], polyurethane [93,94], poly(glycolic acid) (PGA) [87,95], polyethersulfone (PES) [96,97,98,99], and polysulfone [100,101], are more diverse and promising. Some of these materials have been approved by the FDA for clinical human use [49,51,57,102,103,104,105]. Unfortunately, decisions of the FDA may be overturned. This change is associated with a new validation request, which is a long and difficult process. Unlike natural materials, synthetic polymers can be used to produce various shapes of membranes via many techniques and provide cell attachment, as well as good mechanical, physical, and chemical properties that can be modified to improve the parameters of the material. Most of these polymers degrade into components that are metabolized in the body. Moreover, the mechanical properties and degradation time can be controlled by combining these polymers (as copolymers or blends) [20,59,64,74,76,86,102,106,107,108,109,110,111,112,113].

Synthetic materials, like natural materials, have some disadvantages. One of them is an unexpected degradation time, which can cause brittleness of the scaffolds, even during culturing [59]. One example is PLA, which can be influenced by the use of a PLA copolymer with PCL or PEG and will affect the quality of the material [107,110,114]. Synthetic materials also lack desirable biological properties [115]. Moreover, the degradation products can result in side-effects for the host organism. These side-effects mostly involve acids that can be toxic to cells during cultivation or even elicit an inflammatory response in the host organism [59,86,103,116].

To date, studies have been conducted to obtain hybrid materials. Hybrid scaffolds combine the advantages of both synthetic and natural materials, allowing one to obtain membranes with defined mechanical properties featuring the retained bio-functionality and tunable degradation necessary for the regeneration of cartilage [51,57,58,115].

### 3.3. Methods for Obtaining Scaffolds

**Figure 4:**
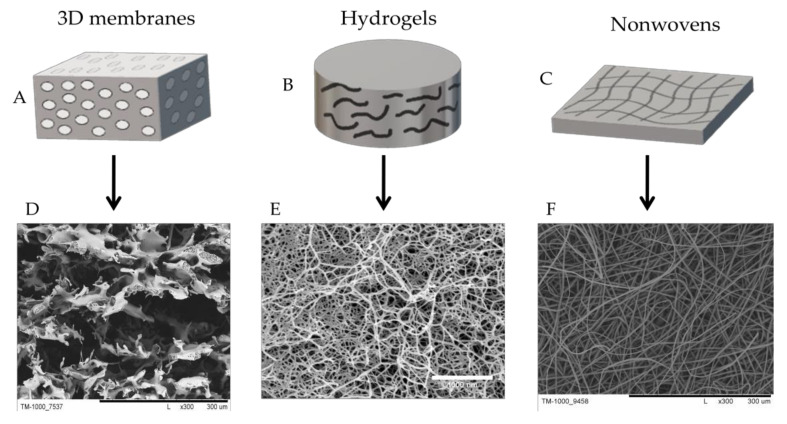
Schematic illustration of the main forms of scaffolds for cartilage tissue engineering: (**A**,**B**) hydrogels; (**C**,**D**) sponges; (**E**,**F**) nonwoven (nanofibers). Scale bars: D—300 µm; E—1000 nm; F—300 µm.

The desired architecture, mechanical parameters, and forms of a scaffold can be obtained by selecting appropriate scaffold production methods. Scaffolds can be formed into 3D membranes (sponges), hydrogels, nonwovens (nanofibers) (Figure 4), or combinations thereof.

The literature describes many methods for producing a network of connected pores offering control over the mechanical properties and the time of scaffold degradation. This section will present the most popular techniques for obtaining synthetic and hybrid scaffolds for cartilage tissue engineering [17,19,21,40,59,86,117,118].

One of the most frequently used methods is phase inversion. Depending on the factor that induces the phase separation of the polymer solution, phase inversion can be carried out in two ways. One such method involves the temperature. This method is called thermal-induced phase separation (TIPS) and can be performed in liquid–liquid and liquid–solid systems, where the temperature of the process is appropriately selected. This method can obtain large and small pores with different membrane porosities [59,66,119,120,121]. In the second case, the phase inversion factor is non-solvent. This is the so-called non-solvent induced phase separation (NIPS) method. Here, the properly formed polymer solution is immersed into a non-solvent of the polymer. The phase inversion then produces a membrane. In these cases, like with the TIPS method, membranes with different porosities and pore sizes can be obtained [122,123,124]. A variant of this method involves adding a pore precursor to a previously prepared polymer solution or during the formation of a membrane. This approach promotes the formation of larger pore sizes and higher porosity. Pore precursors are ultimately removed from the scaffold by an appropriate solvent (porogen-leaching) [71,97,117,118,125,126,127]. Another similar method is solvent-casting particulate leaching (SCPL). This method involves dispersing salt particles in a biocompatible polymer solution. The solvent used to dissolve the polymer is then evaporated to obtain a polymer/salt composite membrane. Then, the salt is leached out by dipping the membrane into water or other salt solvents (not polymer solvents). The obtained membrane is dried to produce a porous compatible membrane. The most commonly used pore precursors are sodium bicarbonate, sodium chloride, and a sodium acetate preparation of polycaprolactone [128].

The freeze-drying technique is a method that uses the sublimation process. In the first step, the polymer is dissolved in a suitable solvent, and then the polymer solution is cooled to its freezing point. In this way, by means of sublimation, the solid solvent is evaporated to obtain a scaffold with multiple pores. With this technique, the dissolved substances can be separated in the ice phase and a small porous structure can be obtained. The final scaffolds are then formed after the final drying. The advantage of this method is its application in biomedical contexts due to the use of water and ice crystals instead of organic solvents in the preparation of the scaffolds. One is also able to control the sizes of the pores by changing the freezing method. The disadvantages of this method are its high energy consumption and a long preparation process [21,59,117,118].

Another common method used to obtain scaffolds is electrospinning. This is a simple and effective technique that can obtain nonwovens from both natural and synthetic polymers. In this method, electrostatic forces are used to produce fibers or spheres with different morphologies and sizes at the micrometer and nanometer scales. Electrospun scaffolds can be characterized by their high porosity, good mechanical properties, and flexibility [117,129,130,131,132]. Moreover, the fibers can be modified using this method, e.g., by functionalizing the fiber surface via enzyme immobilization [133,134]. Therefore, the fibers obtained by the electrospinning technique have wide biomedical applications in addition to their use as scaffolds in tissue engineering [129,130,131,135]. 

The aforementioned methods are considered conventional. A group of more advanced methods, known as rapid prototyping (RP) techniques, enable the production of three-dimensional objects with precise spatial control over the polymer structure. In this way, scaffolds can be obtained gradually, layer by layer, according to computerized data, such as computer-aided design (CAD) or computed tomography (CT) data [5,59,136]. The most common and popular RP techniques involve 3D printing (3DP). As mentioned above, 3DP consists of creating tools and prototype functions directly from computer models. This technique is carried out by applying powdered material in layers and selectively fusing the powder through “inkjet printing” the adhesive. Then, after the layers are deposited, the unbound powder is removed and a 3D object is obtained. Three-dimensional printing can be used to precisely control the structures of the scaffolds at the micron level; however, it requires close monitoring of the tissue structure and the mechanical properties of the scaffold. The 3DP technique also includes bioprinting [5,137,138,139,140]. Another RP method is selective laser sintering (SLS), where a laser is the power source used to sinter the powdered material. The advantage of this technique is the excellent control it offers over the microstructures of the obtained scaffolds by adjusting the process parameters, e.g., the percentage composition of the mixed polymer/composite powder blend. Moreover, this method can use ultra-high molecular weights of polyethylene. Unfortunately, in this process, an additional procedure is required to remove the injected powder, which, in addition to its high operating temperature, is the main disadvantage of SLS [5,141]. In addition to RP, relevant methods include stereolithography (SLA) [142,143,144] and fused deposition modeling (FDM) for creating an object through the controlled deposition of molten material [5,145,146].

The scaffold material, biological factors, and even cells can be used in RP methods. These elements make it possible to obtain a construct with a precise, controllable, and complex internal structure featuring appropriate mechanical properties [59,118,136,137,147,148]. A combination of the above methods can obtain hybrid membranes, which are important due to the sensitivity of some materials to technological conditions, such as temperature. This approach can obtain scaffolds with appropriate mechanical properties as well as appropriate biological parameters. For example, it is possible to combine electrospinning techniques with 3D printing [118,149,150,151].

Cross-linking should also be mentioned as a common method used for the preparation of hybrid scaffolds. Cross-linking can be done in essentially two ways: (1) the formation of a multi-functional molecule with a low molecular weight, resulting in higher molecular weight branched structures and, ultimately, continuous cross-linked structures, and (2) obtaining a networked structure by bonding long linear polymer molecules. Cross-linked polymers have properties that give them numerous applications, including their (1) resistance to solvents, (2) common high softening and heat-distortion temperatures, and (3) excellent dimensional stability. In freeze-drying and cross-linking techniques, it is possible to cross-link polymers during freeze-drying fabrication. [152,153,154]. Cross-linking polymerization is commonly used to produce hydrogels using hydrophilic monomers with cross-linkers. The components can be of both natural and synthetic origin [155]. Table 2 presents the advantages and disadvantages of the methods for obtaining scaffolds.

## 4. Scaffolds for Cartilage Treatment

Scaffolds for cartilage regeneration can be made of synthetic or natural polymers or acombination of both (a hybrid scaffold). Commercial scaffolds for regenerative cartilage are mainly made of natural materials, such as collagen or hyaluronic acid [40,44,156]. Due to the disadvantages of natural materials, research is underway to obtain scaffolds from synthetic and hybrid materials with the addition of biological components—membranes where the advantages of both synthetic and natural materials are taken into account [40,51,64]. This section presents scaffolds made of natural, synthetic, and hybrid materials, along with a short description of them (Table 3). 

### 4.1. Natural Scaffolds

Natural scaffolds are characterized by high bioactivity, biocompatibility, and biodegradability to non-toxic components. Due to their composition of natural materials, these scaffolds are similar to native tissue, which means that their presence creates an ideal environment for cells. Thus, the main advantage of natural polymers is their similarity to cartilage’s ECM components. Their presence appropriately stimulates chondrogenesis and the maintenance of the cellular phenotypes of chondrocytes. These scaffolds affect the adhesion and proliferation of the cell and cell proliferation. Therefore, products used in cartilage regenerative medicine are mainly made of natural materials. Table 4 shows examples of commercial scaffolds, including their materials and basic characteristics. Scaffolds are mostly made of collagen, the main component of cartilage ECM. Unfortunately, these scaffolds often do not meet the necessary requirements, as they quickly lose their structure (sensitivity to an aquatic environment) and transform into a gel-like form. They are also not mechanically strong enough to support the cells and regenerated tissue. Thus, this kind of membrane does not have suitable properties to create hyaline cartilage. As a result of regeneration, non-valuable fibrous cartilage is obtained, which is susceptible to future damage [40,50,51,57,76,111,156,173].

Because natural materials usually have poor mechanical properties, they are often insufficient to regenerate a given tissue. An additional disadvantage is their limited processing methods resulting from the low resistance of the materials to changes in process parameters (e.g., pH, high-temperature, and pressure). For this reason, natural scaffolds do not have the desired parameters. Additionally, the regenerated cartilage is not hyaline cartilage but fibrocartilage with inferior properties.

### 4.2. Hydrogel Scaffolds

Scaffolds of a hydrogel formare of great interest in cartilage regenerative engineering. These scaffolds are formed as a result of the cross-linking of natural and synthetic (or both) polymers and are characterized by their ability to absorb water or biological fluids. All these features make such scaffolds very similar to natural cartilage ECM [155,178,179].

Unfortunately, like natural scaffolds, hydrogel scaffolds have one major disadvantage. Due to their solubility in aquatic conditions, these scaffolds have low mechanical strength, which makes them difficult to handle [105,178]. Intensive studies are currently underway on the development of hydrogels from synthetic and hybrid materials [49,53,176,177,180]. Yang etal. obtained a synthetic hydrogel scaffold with the strength and modulus of native cartilage. This scaffold was composed of a bacterial cellulose (BC) nanofiber network with a PVA–poly(2-acrylamido-2-methyl-1-propanesulfonic acid sodium salt) (PAMPS) double-network hydrogel. BC was chosen as the nanofiber network due to its high tensile strength, biocompatibility, and lack of the enzymes necessary to degrade cellulose in the human body. Moreover, BC mimics collagen. The second layer of the PVA hydrogel provides elasticity, viscoelastic energy dissipation, and tensile resistance by allowing the BC fibers to share the load in the composite framework. This is an example of a scaffold with promising performance for further research in cartilage tissue engineering [180].An example of a hybrid hydrogel scaffold is gelatin/polycaprolactone–polyethylene glycol (Gel/PCEC-TGFβ1) (Table 3).This scaffold uses both natural and synthetic polymers and growth factors. It was prepared and evaluated for human mesenchymal stem cells derived from adipose tissue (h-AD-MSCs). During the study, the tests indicated the expression of cartilage-specific genes, such as collagen type II and aggrecan, showing promising results and potential for further research on cartilage regeneration [165]. 

So-called injectable hydrogels have gained interest in medicine for local deformation in cartilage. In this process, a mixture of the patient’s expanded cells with the hydrogel is injected into the cartilage-damaged area. In the body, cells gradually multiply and the hydrogel is degraded. The advantage of this method is its low invasiveness and the possibility of its precise adjustment to the defect [49,53,179]. There are numerous ongoing/recruiting clinical trials using sealant gel-based MSC constructs for cartilage regeneration [49,176]. Some of them (CARTISTEM^®^, CaReS^®^, and Cartipatch^®^)were approved for clinical usage (Table 4) [49,176,177].

### 4.3. Synthetic Scaffolds 

Research on using synthetic scaffolds for the regeneration of cartilage has been described in numerous studies. These scaffolds are characterized by their biocompatibility, biodegradability, and good mechanical properties and can be obtained by various methods due to their better resistance to physicochemical properties compared to natural membranes. Currently, few synthetic scaffolds are being tested in clinical trials for their use in cartilage regeneration [51].

An example of a commercial scaffold made from synthetic materials is the BioSeed^®^-C (Biotissue) membrane. This membrane is composed of PGA/PLA and PDS materials and is characterized by bioresorbability, elasticity, and the ability to be cut without fraying. This membrane’s adequate 3D structure and the stability of the environment during culturing stimulate the patient’s cells to differentiate [156]. The clinical outcomes at 4 years after implantation showed promising curativeresults for cartilage defects of the knee [157].

The most commonly used synthetic materials for the production of scaffolds for cartilage repair are PLA [66,181,182], PCL [91,159,160,183,184], and copolymers such as PLGA [185,186,187,188] and PLCA [154,163,189]. Christensen et al. used a nanostructured porous polycaprolactone (NSP-PCL) scaffold [159] and compared its in vivo and in vitro outcomes in a rabbit model with a commercial Chondro-Gide^®^ scaffold. The observation time was 13 weeks, and the results were better for the synthetic scaffolds than the commercial ones. This scaffold had higher chondrogenic markers during the in vitro study and better in vivo histological scores. Thus, NSP-PCL seems to be an adequate scaffold for cartilage repair [159]. Research was also conducted with other synthetic materials, such as a spongy PU scaffold [160]. Scaffolds made from PU material had good hydrophilicity and porosity with interconnected pores and adequate mechanical strength. In a previous study, a PU scaffold was compared with a conventional PLA scaffold. The suitability of the scaffold for cartilage regeneration was evaluated during culturing with chondrocytes and human mesenchymal stem cells (MSCs). The chondrocytes grew better and secreted more glycosaminoglycan in the PU scaffolds than in the PLA scaffolds. Moreover, the human MSCs showed greater chondrogenesis in the PU scaffolds than in the PLA membranes. Degradable PU scaffolds thus have potential in cartilage tissue engineering applications [158]. Another example is PES materials. Polysulphonic membranes are an example of scaffolds that offer promising results for the regeneration of cartilage, as these membranes have an interconnected pore network, good elasticity, and excellent mechanical properties [97,98,161,162]. A study with a rabbit model showed that this membrane was better than a commercial Chondro-Gide^®^ scaffold [162]. Unfortunately, the main disadvantage of synthetic polymers is their degradation, which leads to the release of acids. This can cause inflammation in the body. Additionally, in some cases, the degradation is too fast, causing the membranes to break or even crumble; moreover, the membranes do not have adequate biological properties. Therefore, research is being done to obtain scaffolds with a combination of synthetic and natural materials [59,86,103,111,115,116].

### 4.4. Hybrid Scaffolds

Considering the advantages of synthetic and natural materials, scaffolds with good mechanical and biological properties can be obtained. Currently, hybrid scaffolds are one direction of research in pursuit of a suitable implant for articular cartilage regeneration. The membranes of such scaffolds can be improved by inserting other biologically active additives, such as growth factors, and through a selection of appropriate kinds of cells [25,40,49,50,51,64,115,190]. This section presentsand discusses scaffolds made of hybrid materials. Some of these scaffolds are outlined in Table 3 and Table 4.

Currently, few hybrid scaffolds have undergone clinical trials. One of them is Chondrotissue^®^ (Biotissue) [158,166]. This resorbable membrane is composed of PGA and HA and is used in clinical contexts through a one-step treatment method. This membrane’s elasticity is due to the addition of autologous platelet-rich plasma (PRP) or serum enriched with platelets. This method relieves pain, improves mobility, and supports cartilage regeneration. Five years of clinical trials confirmed the good outcomes of this one-step procedure with Chondrotissue^®^, which provides stable results with future potential in hyaline cartilage regeneration.

Rofiqoh et al. developed an IC hybrid scaffold composed of PLGA and collagen. This scaffold features a high porosity membrane with an interconnected pore network and good mechanical properties. The studies were carried out with the use of bovine articular chondrocytes and invivo implantation into mice. The results showed the regeneration of cartilage-like tissue with high potential for further work [153]. Another example of a hybrid scaffold is PLCL-COLI. In a previous study, a PLCL membrane was printed, treated with alkali, and coated with collagen type I (COLI). The obtained scaffold had high porosity with a controlled structure. This scaffold provided good biocompatibility and elastic and mechanical properties. The compressive modulus of the membrane was, moreover, 0.21 MPa (similar to human cartilage). This scaffold provides good outcomes and has promise as an implant in cartilage repair [166]. Next, a C2C1H scaffold was obtained and characterized by Haaparanta et al. This scaffold was composed of collagen, chitosan, and PLA. A synthetic polymer used as a 3D mesh gave the scaffold good mechanical strength, and the natural components mimicked an appropriate environment for chondrocytes. The researchers studied eight scaffolds, determining C2C1H to be the best. This scaffold had a highly porous structure with interconnected pores and good mechanical strength with appropriate stiffness. A culture with isolated bovine chondrocytes showed promising results, with promise for further work towards cartilage regeneration [167]. Another example is the ECM-coated polylactic-co-glycolic acid (ECM-PLGA) scaffold designed by Nogami et al. In this scaffold, a synthetic polymer established the appropriate mechanical properties, while the use of ECM provided an appropriate environment for the cells. This scaffold’s structure achieved the relevant properties for cartilage regeneration. The in vitro study showed attachment, growth, and differentiation of the MSCs. In the invivo study, cell-free scaffolds were implanted into the osteochondral defects of rat knees. Research workers demonstrated that the scaffolds promoted the regeneration of hyaline-like cartilage, which was better than the cartilage in the empty control group. An ECM-PLGA implant may be a good component for use in a one-step method for cartilage regeneration, but more research is required [168,169].

In the literature, there are many examples of hybrid scaffolds used for cartilage tissue engineering, where a biodegradable synthetic polymer provides the housing framework. Mainly, these scaffolds use porous membranes to provide the necessary mechanical properties to support tissue growth, while the additives include natural composites (bioactive fillers). These fillers produce bioactive signals that supply the required information for chondrogenesis and maintain the proper phenotypes of the chondrocytes [25,49,51,110,115,190,191,192]. These are mostly components that naturally occur in the cartilage, such as HA [156,164,193,194], CS [172], COL [64,151,153,167,170,195,196], and ECM [168,197].

## 5. Conclusions

Currently, the most promising method for cartilage regeneration is the transplantation of implants with or without cells in the area with the damage. Therefore, research is underway to obtain an appropriate scaffold. There are many commercial scaffolds used in orthopedics, which, unfortunately, do not completely fulfill their proper roles in the regeneration of hyaline cartilage. New solutions are constantly being sought, including new scaffolds, growth factors, and sources of cells, as well as methods for delivering the implants to damaged areas. Currently, according to the literature, hybrid scaffolds provided the most promising results in research on articular cartilage regeneration. A combination of synthetic materials to ensure adequate mechanical strength and natural components to ensure proper chondrogenesis and preserve the phenotype has the greatest probability of obtaining hyaline cartilage in a damaged area. In addition, the literature provides information on the search for an appropriate method/improvement of current methods for scaffolding production. Boosters that can be added to the scaffolds or the medium (e.g., growth factors) are also being sought. The selection of appropriate types of cells is also under investigation, mainly focusing on MSCs and human autologous chondrocytes. Proper selection of all the above-mentioned factors could ensure that the appropriate articular cartilage regeneration is obtained. The problems to be solved are significant due to the number of people with cartilage problems, such as osteoarthritis. There are several scaffolds designed for orthopedics, but no one solution can guarantee the reconstruction of hyaline cartilage, as most interventions yield fibrocartilage, which is susceptible to further damage. Thus, patients eventually return to the starting point. Consequently, it is important to obtain an appropriate scaffold and method for the regeneration of hyaline cartilage.

Ultimately, two important conclusions can be highlighted. Thus far, no scaffolds have been obtained that achieve the optimal conditions for the regeneration of articular cartilage. The obtained results suggest that due to our poor ability to modify natural materials, hybrid scaffolds and composite ones combining the properties and advantages of several natural and synthetic materials are the most promising options.

## Figures and Tables

**Table 1:** Outerbridge classification of articular cartilage lesions.

Grade of Damage	Description
**Grade 0**	Normal AC with a smooth surface
**Grade I**	Soft and swollen cartilage with a reduced amount of proteoglycans and increased water content.
**Grade II**	The surface is cracked up to half the thickness of the cartilage, a so-called “Blemish” of cartilage. Swelling or fraying is visible via Magnetic Resonance Imaging (MRI) imaging. The area of the damage does not exceed 1.25 cm^2^ (less than 50%) of the surface. This corresponds to damage of an intermediate thickness.
**Grade III**	The damage exceeds half the thickness of the cartilage and may reveal the subchondral bone; the surface of the damage exceeds 1.25 cm^2^. The deep defect comprises more than 50%.
**Grade IV**	Full thickness defect(s). Destruction with complete exposure of the subchondral bone.

**Table 2:** Rapid prototyping (RP) and conventional methods for obtaining scaffolds for tissue engineering cartilage.

Technique	Advantages	Disadvantages
3D printing (3DP)	Possibility of using hydrogels and cells	Low precision Long-standing process Poor mechanical properties
Selective laser sintering (SLS)	Smart process High precision No need for support Construction	High temperature Rough surface
Stereolithography (SLA)	High precision Smart process Soft surface	Risk of high process temperature Untreated Material may be cytotoxic high costs
Fused deposition modeling (FDM)	Good mechanical properties	Poor precision High temperature Narrow range of parameters Limits in application to biodegradable polymers
Bioprinting	High precision Low costs High speed of printing Possibility of supporting high cell viability	Depends on the cell’s existence
Electrospinning	Standard technique for obtaining nanofibrous scaffolds	Toxicity of using solvents Depends on many factors Obtaining 3D structure or/and adequate pore sizes for biomedical applications can be problematic
Freeze-drying	Capability of controlling the pore size Possibility of obtaining high temperatures Used for multiple purposes	Toxicity when using solvents High energy consumption Irregular obtained size pores
Thermal-induced phase separation (TIPS)	Possibility of using a low temperature Very high porosity surface-to-volume ratio Scaffolds obtained from a thermoplastic crystalline polymer	Used only for thermoplastics
Solvent-casting particulate leaching (SCPL)	High porosity Low costs Can be used for fabricating thin membranes of thin-wall 3D specimens	High toxicity when using solvents Time consuming for thin membranes

**Table 3:** Synthetic and hybrid scaffolds for cartilage regeneration.

Scaffold Name [Ref.]	Component	Method	Properties (Porosity (%), Pore Size (µm), Mechanical Properties)	Cell Source/Animal Model	Results
Synthetic scaffolds					
BioSeed^®^-C (Biotissue) [156,157]	PGA/PLA, PDS	Thermoplastic process	Good mechanical properties and adequate structure for cells	Human articular chondrocytes	Assessed in clinical trials. In the results, the scaffolds featured significantly improved final postoperative values. This highlights their effectiveness in cartilage regeneration.
Spongy PU scaffold [158]	PU	Freeze-drying	96.9% 126–186 µm Storage modulus: ~60.36 kPa	Chondrocytes, human MSCs	Biodegradable PU scaffold had better outcomes than PLA 3D membranes during culturing.
NSP-PCL scaffold [159]	PCL	Freeze-drying	The porosity of the scaffold was designed to promote cartilage ingrowth	Rabbit articular chondrocytes	The NSP-PCL scaffold indicated better results during in vitro and in vivo studies compared to the Chondro-Gide^®^ scaffold.
RO45 3DHC [160]	PCL	3D printing	**RO45:** 84.6% 135–285 µm Compressive modulus: 25.6 MPa **3DHC** 83.8% 150–700 µm Compressive modulus: 3 MPa	Human adipose-derived MSCs	The RO45 scaffold was preferable for chondrogenic differentiation compared to 3DHC, which indicated better cell proliferation, scaffold penetration, and more favorable mechanical properties in the final construct.
Polysulphonic scaffold [97,98,161,162]	PES	Non-solvent induced phase separation and porogen- leaching	98.5% 60–300 µm	Rabbit model and human articular chondrocytes	A study with a rabbit model suggested that the scaffold is effective in repairing articular cartilage defects. In vitro study with human cells gave promise results.
PLLA-100 scaffolds [66]	PLLA	Thermally induced phase separation	93% 100 ± 20 μm	Human articular chondrocytes	The scaffold promoted the secretion of chondrogenic genes. It was better than the PLLA scaffold with larger pores (~200 μm).
PLCL-2 scaffold [163]	PLCL	Gel-pressing	80% 300–500 µm Young’s modulus: ~0.7 MPa	Rabbit articular chondrocytes and mice model	The adequate structure of the scaffold showed that chondrocytes did not change their phenotypes during the in vitro study. The in vivo study indicated that the scaffold would maintain mechanical integrity and guide cartilaginous tissue formation.
**Hybrid scaffold**					
Chondrotissue^®^ (Biotissue) [156,164]	PGA, HA	Freeze-drying		Platelet-rich plasma and bone marrow concentrate	The one-step cartilage repair method is available for clinical use. Treatment results follow up to 5 years of good outcomes with the potential for future benefits.
IC scaffold [153]	PLGA, COL	Freeze-drying and cross-linking	99.1% 50–400 µm Young’s modulus: ~9 kPa	Bovine articular chondrocytes (BACs) and mice model	IC scaffold promoted cartilaginous gene expression, chondrocyte proliferation, and the regeneration of cartilage tissue with high mechanical properties. It seems to be promising for cartilage tissue applications.
Gel/PCEC-TGFβ1 hydrogel scaffold [165]	Gelatin, PCEC, TGFβ1	Cross-linking, freeze-drying	~150 μm Young’s modulus: ~0.65 MPa	Human adipose tissue (AD)-MSCs	The study showed the potential for the growth and differentiation of h-AD-MSCs and could be a promising scaffold for cartilage tissue engineering.
PLCL-COLI [166]	PLCL, COL	3D printing	~85% ~10 μm; ~450 μm Young’s modulus: ~0.21 MPa	Rabbit articular chondrocytes	Scaffold with a controlled structure, good biocompatibility, elasticity, and mechanical properties, as well as potential in cartilage regeneration.
C2C1H scaffold [167]	PLA, COL, CH	Freeze-drying and melt-spun	>85% Young’s modulus: 52.3 kPa	Bovine articular cartilage chondrocytes	A hybrid scaffold with high porosity, good mechanical strength, and interconnected pore network. It has potential as a scaffold for cartilage tissue engineering.
ECM-PLGA scaffold [168,169]	PLGA, ECM	SCPL	90%	Rat mesenchymal stem cells (MSCs) and rat model	The in vitro study showed good properties of attachment, proliferation, and differentiation of the MSCs. Involved the implantation of a cell with MSCs and type II collagen mRNA expression. The in vivo study indicated the regeneration of tissue to hyaline cartilage. The scaffold could be promising for cartilage regeneration therapy.
PCL/COL1 [170]	PCL, COL	Selective laser sintering	82.98% Young’s modulus: 3.75 MPa	Pig articular chondrocytes and nude mice model	Scaffold with high porosity and repetitive pore structure. In vitro and in vivo study showed good outcomes compared to the PCL membrane. The addition of collagen ensured the proper development of chondrocytes.
CH/PLLA/PC scaffold [110]	PLLA, CH, PC	Freeze-drying and cross-linking	79–84% 49–170 μm	Rabbit articular chondrocytes	Outcomes from the in vivo study showed the suitability of the scaffold for cartilage tissue regeneration.
Chitosan-modified PLCL scaffold [171]	PLCL, CH	Porogen-leaching, lyophilization, and cross-linking	~85% 200–500 µm Young’s modulus: 0.04 MPa	Pig articular chondrocytes	Biodegradable scaffolds with high porosity, good mechanical strength, and interconnected pore structure. Supplied a good environment for chondrocyte adhesion, proliferation, differentiation, and ECM secretion. The results were good but still require further research.
CSMA/PECA/GO (S2) scaffold [172]	CSMA, MPEG-PCL-AC (PECA), GO		~70% Mean 175.2 μm Compressive modulus: 0.48 MPa	Rabbit articular chondrocytes	Scaffold with an appropriate structure with biological components; provided an adequate environment for cells. The in vivo results were promising with great potential for the future.

CH—chitosan; COL—collagen, PU—polyurethane; PC—pectin based; PDS—poly-p-dioxanone; CS—chondroitin sulfate; CSMA—methacrylated chondroitin sulfate; HA—hyaluronic acid; PEG—poly(ethylene glycol); PCL—polycaprolactone; PLA—polylactic acid; PLLA—poly(l-lactide); PGA—poly(glycolic acid); PES—polyethersulfone; PLGA—polylactic-co-glycolic acid; PCEC—polycaprolactone-polyethylene glycol; ECM—extracellular matrix; PLCL—poly(l-lactide-co-ε-caprolactone); SCPL—solvent casting and particulate leaching method; AC—acryloyl chloride; GO—graphene oxide; PECA—poly(ethylene glycol) methyl ether-ε-caprolactone-acryloyl chloride.

**Table 4:** Natural scaffolds approved for medical use for cartilage tissue engineering.

Product (Company)	Materials	Characteristic
Hyalofast^®^(Anika) [110,154,174,175]	Benzyl ester of hyaluronic acid	A bioresorbable3D scaffold used through a one-step procedure aftera microfracture. It can be used even for deep cartilage lesions. The scaffold’s non-woven structure allows it to be cut and adaptively matched into uneven lesions.
NeoCart^®^(Histogenics) [44,110,154]	Bovine type I collagen	Bioresorbableelectrospun scaffold used in MACI, a two-step procedure. The patient’s chondrocytes are expanded into scaffolds. Then, they are incubated in the Tissue Engineering Processor (TEP), which simulates the variation of mechanical forces and reduces oxygen pressure, allowing the maintenance of the chondrocyte phenotype forming the appropriate proteins of the ECM.
ChondroGide(Geistlich) [110,154]	Type I/III collagen	The first described matrix for the ACI method. It is used in a one-step procedure. ChondroGide’s role is to support and promote the chondrogenic differentiation of MSCs released after the microfracture method.
ACI-Maix^TM^ (MACI) [44,45]	Type I/III collagen	The procedure is a two-step process. Expanded autologous chondrocytes (2 or 3 passage) are cultured into the scaffold for 3 or 4 days before implantation into the patient.
Cartipatch^®^(Xizia Biotech) [44,156,173]	Agarose and alginate	The cylindrical scaffold of a single layer of hydrogel with expanded cartilage cells. The clinical procedure is the same as that for the two-step method. The alginate polymer provides elasticity to the matrix, which facilitates handling during the surgical procedure.
NOVOCART^®^ 3D—AesculapOrthopaedics (BBraun) [44,64,111,156]	Type I collagen, chondroitin sulfate	A sponge scaffold with a bilayer structure and interconnected pores, used in a two-step procedure. This scaffold is desirable in young patients,<16 years old, to avoid eventual secondary injuries, such as early osteoarthritis.
CaReS^®^(Arthrokinetics) [44,64,111,156]	Type I collagen gel	The scaffold is used in a two-step clinical procedure. Isolated autologous chondrocytes are mixed with a fluid matrix. Then, after 14 days, it is set in the lesion using fibrin glue. The height, thickness, and size of the hydrogel can be easily adjusted to the lesion.
CARTISTEM^®^ (Medipost) [49,176,177]	Hyaluronic acid	Allogeneic human umbilical cord blood (hUCB)-derived MSCs and HA hydrogel products for cartilage regeneration for repeated traumas or degenerative osteoarthritis. A 7-year follow-up study of 104 patients showed promising efficacy in terms of durable cartilage regeneration with no significant adverse effects.
